# Visceral Adipose Tissue Depth in Early Pregnancy and Gestational Diabetes Mellitus - a Cohort Study

**DOI:** 10.1038/s41598-020-59065-5

**Published:** 2020-02-06

**Authors:** João Guilherme Alves, Alex Sandro Rolland Souza, José Natal Figueiroa, Carla Adriane Leal de Araújo, Angélica Guimarães, Joel Geoffrey Ray

**Affiliations:** 1 Department of Pediatrics, Instituto de Medicina Integral Prof. Fernando Figueira (IMIP), Rua dos Coelhos, 300, Boa Vista, CEP: 50070-550 Recife, Pernambuco Brazil; 2 Department of Pediatrics, Hospital Dom Malan (HDM), Rua Joaquim Nabuco, S/N, Centro CEP: 56304-900 Petrolina, Pernambuco Brazil; 3 Departments of Medicine and Obstetrics and Gynaecology, St. Michael’s Hospital, 30 Bond St, Toronto, Ontario M5B 1W8 Canada

**Keywords:** Health care, Diagnosis

## Abstract

Some studies have suggested that abdominal visceral adipose tissue depth (VAD) measured by ultrasound in early pregnancy, may predict the future onset of gestational diabetes mellitus (GDM). Wheter this is true, independent of pre-pregnancy body mass index (BMI), has been debated, leading the current study. A prospective cohort study was completed, in which VAD was measured at around 14 weeks’ gestation. GDM was later assessed by an oral glucose tolerance test at 24 to 28 weeks, according to the IADPSG criteria. Logistic regression analysis and receiver operating curve (ROC) analysis were used to estimate the predictive value of VAD, above and beyond pre-pregnancy BMI. 627 pregnant women were enrolled, and 518 completed the study. VAD was measured at a mean of 14.4 weeks’ gestation. 87 women (16.8%) subsequently developed GDM. The unadjusted odds ratio (OR) for developing GDM was 1.99 (95% CI 1.59–2.46) per 1-cm increase in VAD. After adjusting for maternal BMI and age, the OR was 2.00 (95% CI 1.61 to 2.50). The ROC under the curve for developing GDM was higher for VAD (0.70, 95% CI 0.63 to 0.75) than for pre-pregnancy BMI (0.57 95% CI 0.50 to 0.64) (p < 0.001). In conclusion, higher VAD may better predict GDM than pre-pregnancy BMI.

## Introduction

Gestational diabetes mellitus (GDM) – glucose intolerance, resulting in new onset in pregnancy–has emerged as a global public health concern^1^. GDM is associated with potentially harmful health effects for mother and fetus^2^. According to the World Health Organization (WHO), the prevalence of GDM is rising^3^, largely attributed to the worldwide epidemic of obesity, older maternal age at first pregnancy, and a change in the diagnostic criteria for GDM^4^. Currently, the International Association of Diabetes recommends screening for GDM among all pregnant women, at 24 to 28 weeks’ gestation, using a 75-g oral glucose tolerance test (OGTT)^5^. AS this approach lacks a sufficient sensitivity and specificity^6^, a method that may improve the detection of GDM, earlier pregnancy, is desirable.

Some studies have shown that abdominal visceral adipose tissue depth (VAD) measured by ultrasound in earlier pregnancy, may predict glucose intolerance, insulin resistance, metabolic syndrome, newborn weight and GDM later in pregnancy^7–13^. Recently, Thaware *et al*.^14^ showed that ultrasonography‐measured VAD in early pregnancy aided in the early recognition of GDM. They reported that this method, compared with an OGTT alone, could reduce by half number of pregnant women requiring OGTT screening. However, it is not clear whether ultrasonography-measured VAD offers improve discrimination in the detection of GDM compared with simply using pre-pregnancy body mass index (BMI). Herein, we carried out a prospective cohort study among a large sample of pregnant women, to assess the role of VAD in the prediction of GDM.

## Methods

### Design and study participants

This was a prospective cohort study of pregnant women, followed from the first trimester until 24 to 28 weeks’ gestation. The main outcome was development of GDM.

The study was performed at Instituto de Medicina Integral Prof. Fernando Figueira (IMIP), Recife-Brazil, from March 2016 to September 2018. IMIP is a large tertiary maternity hospital with approximately 6,000 deliveries annually. This project was previously approved by IMIP Ethics Committee, and all participants gave written informed consent. The study was developed in accordance with the relevant local guidelines and regulations

Women with a singleton pregnancy at <20 weeks’ gestation, based on last menstrual period or dating ultrasound, were invited to participate. Those with previous history of GDM or pre-gestational diabetes were excluded.

### VAD measurement

VAD was measured by ultrasound by a single sonographer (AR–coordinator of the IMIP service of “Fetal Medicine”, national reference in prenatal care). The Samsung Medison, Accuvix V20 machine was used, according ti the technique by Armellini *et al*.^15^, with a slight modification as described by Martin *et al*.^16^. Intra-observer agreement for VAD has an intra-class correlation coefficient of 0.965 (95% CI 0.960 to 0.985). Measurements were made in supine position, and the 5.2 MHz curvilinear Array probe was placed on the anterior abdomen in the xipho-umbilical line, 1 cm above the umbilicus. VAD was measured as the perpendicular distance between the posterior aspect of the junction of the two rectus abdominis muscles (i.e., the linea alba) and the anterior aspect of the abdominal aorta.

### Diagnosis of GDM

All pregnant women subsequently underwent a 75 g OGTT between 24 and 28 weeks’ gestational week, following a 12-hour fast, with sampling at 1 and 2 hours post-load. Plasma measurements were performed with glucose oxidase methods. A diagnosis of GDM was according to the International Association of Diabetes and Pregnancy Study Group (IADPSG) criteria^17^: fasting concentration ≥5.3 mmol/L, 1-hour ≥10.6 mmol/L, or 2-hour ≥9.0 mmol/L. Those diagnosed with GDM were referred for high-risk antenatal care at IMIP^18^.

### Data analysis

Baseline characteristics of GDM and no-GDM pregnant women were compared using independent samples t-test for continuous variables and chi-squared tests for categorical variables. Logistic regression was performed to assess the risk for GDM per 1-cm unit rise in VAD. Included in the model were maternal age and pre-pregnancy BMI. Receiver operator characteristic curve analyses assessed the predictive value of VAD for new-onset GDM, and Youden index was calculated. Pearson’s correlation analysis was performed to analyze the correlation between VAD and each OGTT value. Statistical analysis was performed using STATA version 12.1; a 5% significance level was considered (p < 0.05).

## Results

Of the 627 pregnant women enrolled, 518 (82.6%) completed an OGTT. Table 1 shows the characteristics of those who did and did not complete the OGTT. VAD was measured at a mean of 14.4 weeks’ gestation.

**Table 1:** Characteristics of pregnant women who completed the study and those lost to follow-up.

Characteristic	Completed the study (n = 518)	Lost to follow-up (n = 109)	p-value
Age, years)	26.1 (5.8)	26.2 (5.8)	0.834
Pre-pregnancy body mass index, Kg/m^2^	26.0 (5.1)	25.5 (4.9)	0.211
Gestation age at entry, weeks	13.5 (1.1)	13.6 (1.1)	0.627
Number (%) with ≥2 prior births	208 (40.1%)	66 (39.8%)	0.891
Educational attainment, years	11.3 (2.1)	11.4 (2.1)	0.723

All data are shown as a mean (SD) unless otherwise specified.

The mean (SD; 95% CI) VAD by ultrasound was 5.44 (±1.27) cm; 95% CI 5.33–5.55. 87 (16.8%) pregnant women developed GDM according to IASDPG criteria. VAD, age and BMI pre-pregnancy differed between women who did and did not develop GDM (Table 2).

**Table 2:** Baseline characteristics of women according to their diagnosis of gestational diabetes mellitus in the index pregnancy.

Measure	No Gestational Diabetes Mellitus (n = 431)	Gestational diabetes mellitus (n = 87)	p-value
Visceral adiposity depth, cm	5.2 ± 1.1	6.3 ± 1.3	0.001
Gestational age at visceral adiposity depth measurement, weeks	14.4 ± 3.0	14.1 ± 3.2	0.4
Age, years	26.0 ± 5.7	27.5 ± 5.8	0.02
Number (%) with ≥2 prior births	169 (39.3%)	39 (44.8%)	0.3
Pre-pregnancy body mass index, kg/m^2^	24.4 ± 4.5	25.4 ± 4.6	0.04
Educational attainment, years	11.4 ± 2.1	11.4 ± 1.6	0.96
Number (%) smoker	10 (2.3%)	1 (1.1%)	0.5

All data are shown as a mean (SD) unless otherwise specified.

There was a significant correlation between VAD and the OGTT fasting glucose (r = 0.179, 95% CI 0.094 to 0.261; p < 0.001), 1-hour glucose (r = 0.238, 95% CI 0.154 to 0.319; p < 0.001) and 2-hour glucose (r = 0.221, 95% CI 0.136 to 0.303; p < 0.001) concentration.

There was a significant relation between VAD and subsequent development of GDM (OR = 1.99, 95% CI 1.59–2.46; p < 0.01) (Table 3). The association remained statistically significant after adjusting for maternal age and pre-pregnancy BMI (OR 2.00, 95% CI 1.61 to 2.50).

**Table 3:** Odds ratios for developing gestational diabetes mellitus in relation to visceral adipose tissue depth measured by ultrasonography at about 14 weeks’ gestation.

Measure	Unadjusted odds ratio (95% confidence interval)	Adjusted odds ratio^a^ (95% confidence interval)
Visceral adipose tissue depth (per 1-cm unit increase)	1.99^*^ (1.59–2.46)	2.00^**^ (1.61 to 2.50)

*p = 0.001; **p = 0.001.

^a^Adjusted for pre-pregnancy body mass index (continuous, in kg/m^2^) and maternal age (continuous, in 1-year increments).

**Figure 1:**
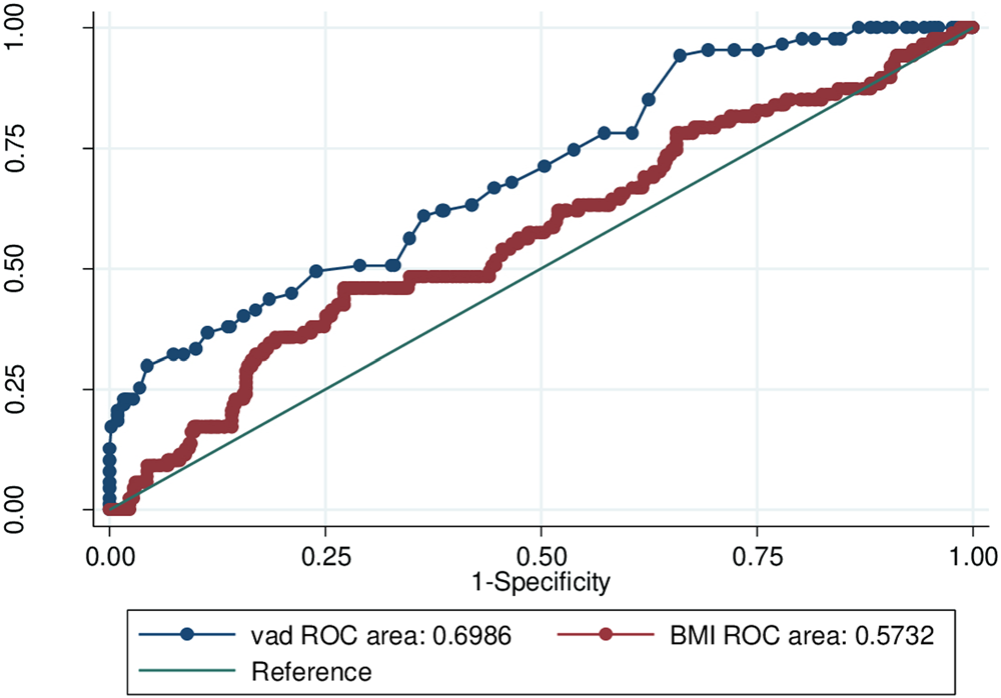
Receiver operator curves for visceral adipose tissue depth (VAD) and pre-pregnancy body mass index (BMI), and their respective associations with developing gestational diabetes mellitus.

The ROC curve analysis of GDM showed a higher under the curve for VAD (0.70 95%CI 0.63 to 0.75) than pre-pregnancy BMI (0.57 95% CI 0.50 to 0.64) (p < 0.0001 (Fig. 1). The optimal VAD cut off for that maximized Youden’s index was 5.1 cm.

## Discussion

An increasing VAD, measured by ultrasonography in early pregnancy, was associated with higher risk of GDM. This risk persisted after adjustment for pre-pregnancy BMI. Furthermore, ROC analysis showed VAD had a significantly higher predictive area under curve for developing GDM than did pre-pregnancy BMI.

The observed association between VAD in early pregnancy and a higher risk of GDM is consistent with other studies. However, our study also shows the superiority of first trimester VAD over pre-pregnancy BMI.

The first description of the association between visceral adiposity in early gestation and glucose intolerance in later pregnancy was made in 2009 by Martin *et al*.^7^. In 62 pregnant women, they observed that a VAD above the upper quartile value was associated with an increased risk for a positive glucose challenge test between 24 and 28 weeks’ gestation. De Souza *et al*.^8^ completed a prospective cohort study of 79 pregnant women and observed that VAD, measured by ultrasonography at 11 to 14 weeks’ gestation, explained 42% of the variance in HOMA-IR. In another prospective cohort of 485 women, De Souza *et al*.^9^ showed that an elevated VAD, assessed by ultrasound at 11 to 14 weeks’ gestation, independently predicted the risk of dysglycemia later in pregnancy. However, Pontual *et al*. observed that VAD, measured in the first half of pregnancy, was no better than pre-pregnancy BMI in predicting insulin resistance and dyslipidemia later in pregnancy^16^.

In a cohort of 1048 pregnant women, Bourdages *et al*.^10^ observed that first-trimester VAD was associated with a higher likelihood of developing GDM. They also reported a marginal improvement in GDM prediction using VAD, over using BMI alone. Recently, D’Ambrosi *et al*.^11^ showed that VAD measured at 24–28 weeks’ gestation, was higher in cases with GDM than controls with no GDM. VAD may be superior to BMI in predicting GDM, as BMI does not reflect the metabolically actively visceral adipose tissue compartment to the same degree as does VAD. Moreover, VAD, measured by ultrasound, is a safe and simple method to carry out in early pregnancy, such as at the time of pregnancy dating or at the determination of fetal nuchal thickness^7–9^.

This study has several strengths. It prospectively included a large sample of women, using a standardized protocol. IADPSG criteria were used to diagnose GDM. All sonographic exams were performed by an single ultrasonographer, previously trained in the technique of Armellini *et al*.^14^. This study also has some limitations. Pregnant women entered the study in the end of the first trimester of pregnancy, once some metabolic and habitus changes would have occurred. During follow-up, 17.4% of participants left the study. The worsening economic crisis in Brazil during the data collection phase, along with the onset of the Zika virus epidemic, likely contributed to loss of follow-up. Even so, the clinical characteristics of those who did and did not complete the study were similar.

In conclusion, there was an increased risk of GDM in relation to VAD measured in early pregnancy. This association remained so after adjusting for BMI, and VAD was more predictive of GDM than pre-pregnancy BMI. Further studies are needed to better explore whether a reduction in VAD depth can reduce the subsequent risk of GDM, whole improving perinatal outcomes.

## References

[1] Guariguata L, Linnenkamp U, Beagley J, Whiting DR, Cho NH (2014). Global estimates of the prevalence of hyperglycaemia in pregnancy. Diabetes Res. Clin. Pract..

[2] Reece EA (2010). The fetal and maternal consequences of gestational diabetes mellitus. J. Matern. Fetal Neonatal Med..

[3] World Health Organization. Global report on diabetes https://appswhoint/iris/bitstream/10665/204871/1/9789241565257_engpdf (2016).

[4] Hunt KJ, Schuller KL (2007). increasing Preval. diabetes pregnancy Obstet. Gynecol. Clin. N. Am..

[5] American Diabetes Association Standards of medical care in diabetes—2017 Am Diabetes Assoc, **40** (Suppl. 1), S18 (2017).

[6] Lovati E (2013). Gestational diabetes mellitus: including serum pregnancy-associated plasma protein-A testing in the clinical management of primiparous women? A case-control study. Diabetes Res. Clin. Pract..

[7] Martin AM (2009). Abdominal visceral adiposity in the first trimester predicts glucose intolerance in later pregnancy. Diabetes Care.

[8] De Souza, L.R. *et al*. Abdominal adiposity and insulin resistance in early pregnancy. *J Obstet Gynaecol Can* **36**969–975 (2014). 10.1016/S1701-2163(15)30409-6 25574673

[9] De Souza, L. R. *et al*. First-Trimester Maternal Abdominal Adiposity Predicts Dysglycemia and Gestational DiabetesMellitus in Midpregnancy. *Diabetes Care* **39**61–64 (2016). 10.2337/dc15-2027 26525976

[10] Bourdages M (2018). First-Trimester Abdominal Adipose Tissue Thickness to Predict Gestational Diabetes. J. Obstet. Gynaecol. Can..

[11] D’Ambrosi F (2018). Maternal Subcutaneous and Visceral Adipose Ultrasound Thickness in Women with Gestational Diabetes Mellitus at 24–28 Weeks’ Gestation. Fetal Diagn. Ther..

[12] Cisneiros RM (2013). Visceral adiposity in the first half of pregnancy predicts newborn weight among adolescent mothers. J. Obstet. Gynaecol. Can..

[13] Gur EB (2014). Ultrasonographic visceral fat thickness in the first trimester can predict metabolic syndrome and gestational diabetes mellitus. Endocr..

[14] Thaware PK, Patterson CC, Young IS, Casey C, McCance DR (2019). Clinical utility of ultrasonography-measured visceral adipose tissue depth as a tool in early pregnancy screening for gestational diabetes: a proof-of-concept study. Diabet. Med..

[15] Armellini F (1990). The contribution of sonography to the measurement of intra-abdominal fat. J. Clin. Ultrasound.

[16] Martin. A (2009). Abdominal Visceral Adiposity in the First Trimester Predicts Glucose Intolerance in Later Pregnancy. Diabetes Care.

[17] World Health Organization. Diagnostic criteria and classification of hyperglycaemia first detected in pregnancy: a World Health Organization guideline. Available from: http://apps.who.int/iris/bitstream/10665/85975/1/WHO_NMH_MND_13.2_eng.pdf. Accessed 11 November (2016)

[18] Pontual AC, Figueiroa JN, De Souza LR, Ray JG, Alves JG (2016). Visceral Adiposity in the First Half of Pregnancy in Association with Glucose, Lipid and Insulin Profiles in Later Pregnancy: A Cohort Study. Matern. Child. Health J..

